# Investigating Rhythmicity in App Usage to Predict Depressive Symptoms: Protocol for Personalized Framework Development and Validation Through a Countrywide Study

**DOI:** 10.2196/51540

**Published:** 2024-04-24

**Authors:** Md Sabbir Ahmed, Tanvir Hasan, Salekul Islam, Nova Ahmed

**Affiliations:** 1 Design Inclusion and Access Lab North South University Dhaka Bangladesh; 2 Department of Computer Science and Engineering United International University Dhaka Bangladesh

**Keywords:** depressive symptoms, app usage rhythm, behavioral markers, personalization, multitask learning framework

## Abstract

**Background:**

Understanding a student’s depressive symptoms could facilitate significantly more precise diagnosis and treatment. However, few studies have focused on depressive symptom prediction through unobtrusive systems, and these studies are limited by small sample sizes, low performance, and the requirement for higher resources. In addition, research has not explored whether statistically significant rhythms based on different app usage behavioral markers (eg, app usage sessions) exist that could be useful in finding subtle differences to predict with higher accuracy like the models based on rhythms of physiological data.

**Objective:**

The main objective of this study is to explore whether there exist statistically significant rhythms in resource-insensitive app usage behavioral markers and predict depressive symptoms through these marker-based rhythmic features. Another objective of this study is to understand whether there is a potential link between rhythmic features and depressive symptoms.

**Methods:**

Through a countrywide study, we collected 2952 students’ raw app usage behavioral data and responses to the 9 depressive symptoms in the 9-item Patient Health Questionnaire (PHQ-9). The behavioral data were retrieved through our developed app, which was previously used in our pilot studies in Bangladesh on different research problems. To explore whether there is a rhythm based on app usage data, we will conduct a zero-amplitude test. In addition, we will develop a cosinor model for each participant to extract rhythmic parameters (eg, acrophase). In addition, to obtain a comprehensive picture of the rhythms, we will explore nonparametric rhythmic features (eg, interdaily stability). Furthermore, we will conduct regression analysis to understand the association of rhythmic features with depressive symptoms. Finally, we will develop a personalized multitask learning (MTL) framework to predict symptoms through rhythmic features.

**Results:**

After applying inclusion criteria (eg, having app usage data of at least 2 days to explore rhythmicity), we kept the data of 2902 (98.31%) students for analysis, with 24.48 million app usage events, and 7 days’ app usage of 2849 (98.17%) students. The students are from all 8 divisions of Bangladesh, both public and private universities (19 different universities and 52 different departments). We are analyzing the data and will publish the findings in a peer-reviewed publication.

**Conclusions:**

Having an in-depth understanding of app usage rhythms and their connection with depressive symptoms through a countrywide study can significantly help health care professionals and researchers better understand depressed students and may create possibilities for using app usage–based rhythms for intervention. In addition, the MTL framework based on app usage rhythmic features may more accurately predict depressive symptoms due to the rhythms’ capability to find subtle differences.

**International Registered Report Identifier (IRRID):**

DERR1-10.2196/51540

## Introduction

### Background

#### Need for Identification of Depressive Symptoms

Every 40 seconds, a person commits suicide and there are more than 20 attempts worldwide [1]. Among suicide attempters, major depressive disorder (MDD) is common [2], and people having MDD are at greater risk of suicidality [3]. Despite these facts, there is a significant lack of interventions to mitigate depression, due to which the depression rate is increasing. In fact, it is estimated that depression will rank first as a global burden of disease by 2030 [4]. In addition, due to the COVID-19 pandemic, a significantly higher number of people have MDD [5], and such a negative impact may persist for a prolonged period. In Bangladesh, the depression prevalence is higher than the overall rate in South Asia [6]. Compared to Bangladeshi people of any other professions, the depression rate is higher among university students [7] (eg, 82.4% of students have at least mild depression [8]), which is alarming.

To significantly facilitate early interventions to mitigate depression, there is an urgent need for its early identification [9]. A person is identified as depressed if symptoms (eg, hopelessness) appear for a period, such as most days for a minimum of 14 days [10,11]. However, it is difficult to precisely assess depressive symptoms [12]. In fact, primary care providers fail to identify depression in more than 50% of cases [13,14]. Understanding the depressive symptoms, such as those in the 9-item Patient Health Questionnaire (PHQ-9) [15], of a person in real time can significantly facilitate mental health care professionals to understand the illness more precisely, to identify depression early, and to take steps accordingly for intervention.

#### Pervasive Health Research in Low- and Middle-Income Countries

Although over 80% of the burden of depression is found in low- and middle-income countries (LMICs) [16], there remains a severe scarcity of mental health care professionals in LMICs. In Bangladesh, there are only 565 psychologists [6], although the population is over 165 million [17], with 1.234 million university students [18]. In these cases, a pervasive technology, such as a smartphone-based monitoring system, which is available to a large number of people in LMICs, can play a significant role [19] In addition, to minimize the barriers to health care facilities in low-resource settings, artificial intelligence (AI)–based mobile apps can be useful [20]. However, almost all the studies that have demonstrated a pervasive technology–based automated system to identify depression have been conducted in the context of high-income countries. For instance, all the studies included in a recent systematic review were from countries other than LMICs [21], which indicates how little pervasive health research has been conducted in the context of LMICs. As a result, the models developed based on participants from high-income countries may not be applicable in LMICs, since the behavior (eg, app usage [22]) varies among countries and various factors, such as socioeconomic status [23] and culture [22], impact behavior.

#### App Usage Rhythm That May Resemble Biobehavioral Rhythm

Zeitgebers, social and environmental cues, help a person’s rhythms synchronize well [24], which can impact their daily activities. Rhythms based on pervasive device–sensed physiological data change depending on external cues, such as light exposure, eating time, and physical activity [24]. Similarly, smartphone usage behavior is linked with factors such as eating behavior [25]. In addition, there is a relation of app usage with alertness, chronotype [26], and physiological data, such as sleep [26-28]. Like physiological data, app usage behavioral markers vary depending on the hour of the day [29-31] and exercise [28]. These facts show app usage behavior may also have a rhythmic pattern with reproducible waveforms similar to the rhythms based on physiological data.

Although a recent study extracted parametric rhythmic feature–dominant periods from smartphone usage data [32], the study was limited by not exploring rhythmic features, such as the acrophase, interdaily stability (IS), intradaily variability (IV), and relative amplitude (RA). In addition, the study explored mere screen usage, without any exploration of more informative features [33], such as entropy data–based features. In the case of other previous app usage data–based studies, researchers used descriptive statistical methods [29-31] to determine whether there is any variation over the day and inferential statistical methods [34,35] to find the difference in terms of aggregated data of the 4 time periods, namely morning, afternoon, evening, and night. These approaches have some limitations. First, there is a lack of statistical evidence to show whether there exists any rhythm that could be resolved with the zero-amplitude test [36,37], which is used to detect rhythm in the field of chronobiology. In addition, the mere analysis in presenting the difference between the aggregated data of morning and evening cannot show whether there is a cycle that can repeat over days. The average data may also miss the microscopic view of data [38]. However, an analysis fitting mathematical models (eg, cosinor model) to time series data can present a microscopic view of the data and inferential statistical estimates of the rhythmic properties [38]. In addition, many informative behavioral markers, such as the dominant period, stability in behavior, and the peak time of oscillations in the rhythm, cannot be extracted from just finding the differences between periods (eg, morning vs night).

#### Potential of App Usage–Based Rhythms in Identifying Depressive Symptoms in LMICs

In human life, physiological changes reappear in a cyclable waveform [38]. Rhythm features based on physiological data have been explored in both the chronobiology [39] and pervasive health [32] areas. Researchers have found a relation between physiological data–based rhythmic markers and health status [32]. These markers can find out subtle differences that enable marker-based features to predict hospital readmission [40] and to identify loneliness and the depression class [32]. This shows the possibility of improving the performance of the models upon the incorporation of app usage rhythmic features to predict the symptoms of depression. However, previous studies [32,40] have mostly relied on wearables to extract rhythmic features, which are costly and may not be affordable [41] for people with a low income. In contrast, smartphones are economically attainable [42], and app usage data are resource insensitive [33]. As a result, app usage data–based systems may be feasible in LMICs.

#### Predicting Depression and Depressive Symptoms Through an Unobtrusive Method

##### Classification of the Depressed and Nondepressed

Most existing research based on AI for mental health has worked on classification problems [43]. Researchers have classified depressed and nondepressed individuals by developing personalized models [44] and using contextually filtered features where the rule mining technique was incorporated [45]. Some other studies (eg, [46,47]) have relied on sensing data (eg, GPS data), along with smartphone data call history, to predict depression. Researchers have also leveraged internet usage data [48], location data retrieved through the campus WiFi infrastructure [49,50], and the GPS [50-52]. Recently, some researchers focused on exploring rhythmic features to assess depression. For instance, Yan et al [32] leveraged rhythm-based features to classify depressed and nondepressed participants. However, the classification does not provide precise information about a participant’s depressive status, since scores of all the symptoms are aggregated to keep the participant in a particular group (eg, depressed or moderate depression), resulting in a loss of the complexity of the psychological problem of depression.

##### Predicting Depression Scores

Compared to classification research problems, there is relatively less research on predicting the depression score (eg, PHQ-9 score=11). Studies in the pervasive health area can be broadly categorized into those that have developed models leveraging data based on both smartphones and wearables [53-56]; only smartphones [53,55]; various sensing devices, along with social media [57]; and subjective and smartphone data [58]. There remain mixed findings on whether the smartphone has superior performance than the wearable. Smartphone-sensed data showed higher performance in a previous study [55] when models were evaluated after splitting training and testing data based on time. However, in the same study [55], researchers found smartphones have inferior performance on another evaluation criterion. Regardless of superiority, both wearables and smartphones show promising performance in the automated assessment of depression [53], which can play a role in real-time remote monitoring of depressed individuals [59]. Wearable- and smartphone-sensed behavioral markers, such as call duration [58] and heart rate [60], as well as inferred markers, such as the circadian rhythm [60], have a significant correlation with the depression score, which may explain the fact of enabling the sensed data to predict depression. However, like the classification, in the prediction of depression scores also, there remains the inability to precisely understand depressive symptoms since a person’s depression score (eg, 11) can be found in combination with different frequencies of the appearance of different symptoms.

##### Predicting Symptoms and Multitask Learning

Researchers have conducted a network analysis of depressive symptoms and presented a possible viable target for intervention [61] and central symptoms for possible focused treatments [62]. The relation of ecological momentary assessment of depressive symptoms with the PHQ-9 score [63] and the relation of depressive symptoms with behavioral data [64] have also been investigated in previous studies. Exploring pervasive device–sensed data, researchers have found a link of higher spending duration in students with higher fatigue, which is a depressive symptom [64]. Although there are studies predicting symptoms of other psychological problems, such as schizophrenia [65] and attention-deficit/hyperactivity disorder (ADHD) [66], few studies [67] have predicted the appearance of depressive symptoms. In a previous study [67], authors predicted depressive symptoms through the data of iPhone users and Android users. However, their study has several limitations. First, their models’ performance is low in the case of the maximum depressive symptoms; particularly, the specificity score is around 60% or below 60% in many models developed by leveraging smartphone data. Second, they developed a separate model to predict each symptom, which makes the model resource sensitive. Third, they considered each symptom-predicting task as a separate one, which could be improved by leveraging the shareable information among symptoms through the multitask learning (MTL) framework as MTL has superior performance than the single-task learning (STL) technique [53,65]. However, researchers have grouped all the predicting tasks into a single model in previous studies [53,65], which may lower performance since all tasks do not help each other to improve performance [68].

### Objective

To overcome the above-mentioned limitations, the main objective of this study is to explore app usage data–based rhythmicity detection and develop and validate an MTL framework leveraging app usage data–based parametric and nonparametric rhythmic parameters through a countrywide study in Bangladesh. Another objective of this study is to explore whether app usage rhythmic features are related to depressive symptoms.

## Methods

### Ethical Considerations

**Figure 1:**
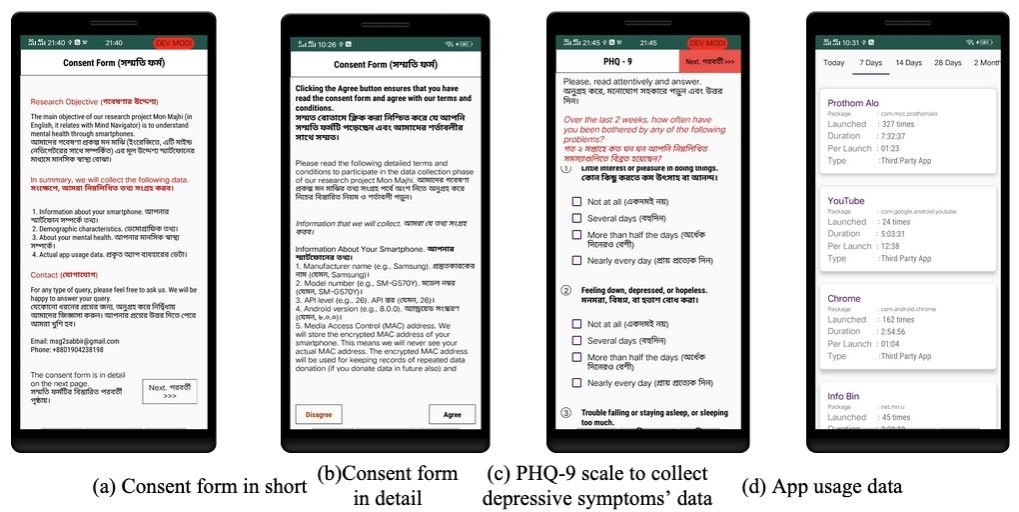
Screenshot of the data collection tool showing the (a) consent form in short in Bangla and English, (b) consent form in detail, (c) PHQ-9, and (d) app usage data. PHQ-9: 9-item Patient Health Questionnaire.

Our study was approved (reference: 2022/OR-NSU/IRB/0704) by the North South University Institutional Review Board/Ethics Review Committee. While collecting data, we presented the consent form in short in Bangla (native language) and English (Figure 1a), where we provided a summary of the data that we collected. In addition, we provided a consent form in detail (Figure 1b) about different matters, including data safety, privacy, and each data item that we collected. Furthermore, before data collection, we briefly described the research to the participants. All participants provided their consent. After collecting data, we provided an incentive of food tokens equivalent to around US $0.30 to almost all participants. For certain participants (<5%) where it was not feasible to give food tokens, we provided an equivalent gift in other forms (e.g., by book diary).

All participants’ data are stored in the Google Firebase database, which only 1 of the researchers can access with 2-factor authentication. We did not collect any personal information from the participants, such as name, email, and phone number. In addition, we did not collect the names of the participants’ departments and universities so that they would feel more comfortable in providing data. We reported the number of departments and universities based on our aggregated notes.

### Retrieval of App Usage Behavioral Markers

In this study, we will focus on developing our system in such a way that the users of the system and also health care professionals can be informed about depressive symptoms in real time. For instance, a health care professional, with the user’s consent to access information, can be notified if our system’s automatic prediction in a day shows that the depressive symptoms can worsen. For this research, we used our previously developed app [69], which can retrieve each participant’s past 7 days’ foreground and background app usage event data within 1 second once the participant provides consent. The average time required to retrieve app usage events is 307.94 milliseconds (SD 1.1 seconds) [33]. For each app usage event, there are data on the app name, package name, and timestamp of the event, which we will use to extract behavioral markers. The app (Figure 1) was used in our previous studies to explore different research problems, including students’ academic results [70-72], depression [33-35,73], and loneliness [74,75], showing the app’s reliability and validity.

### Sample Size Determination and Data Collection

In Bangladesh, there are around 1.234 million university students [18], and an optimal sample that represents the behavior of this population with a 95% CI and a 5% margin of error is 385 university students, as we found with SurveyMonkey [76], which uses the formula for the finite sample size [77]. Using the same formula, we found that the required sample size to represent the 0.448 million female and 0.786 million male student groups each is 384 [18]. Since the depression rate differs between students of public and private universities in Bangladesh [78], we collected data from both types of universities. In the 0.329 million and 0.902 million students of public and private universities, respectively [18], we found that the required sample size in each case is 384. However, there is no fixed sample size that can ensure the generalizable performance of machine learning (ML) models. Therefore, to develop an impactful model, we tried to maximize the number of participants by conducting a countrywide study. In addition, we tried to maximize the number of students from public universities because the largest number of students study there [18].

We collected data from all 8 divisions of Bangladesh using the multistage convenient sampling method. From each division, we collected data from at least 1 university and multiple departments. We tried to maximize the diversity among the participants because the socioeconomic status and many other demographic characteristics vary by region of a country, which can have an impact on mental health [78] and smartphone usage behavior [79,80].

We collected data from a total of 2952 participants from September 2022 to March 2023. While collecting data, first, through our app [69], the participants responded to questions about demographic characteristics (eg, gender) after providing consent. Next, using the same app, they responded to the items of the PHQ-9 [15] based on their experiences of the past 14 days. After saving the responses to all psychological questions, the app automatically retrieved their app usage data, which may take less than 1 second in almost all cases, as shown in our previous estimation [33]. After retrieval, the app saved the app usage data instantly.

### Data Preprocessing and Data Set Description

Our app could not retrieve any app usage data from 14 (0.47%) participants’ phones. One of the plausible reasons for this problem could be system problems in the phones, as shared by the participants. Several of these participants shared that they do not use the original version of the phones. In addition, for 2 (0.07%) participants, age values were missing, and we did not impute these values, as age information was not required for the primary purpose of the research. Furthermore, data on 82 (2.77%) participants’ professions were missing. We imputed the missing professions based on 2 pieces of information: First, in our study, only 2 (0.07%) participants were not students, and we did not reach out to them for data collection; they provided data by installing the app from Google Play Store, which was based on their own interests, indicating a low probability of having a profession other than “student.” Second, all 82 (2.77%) participants provided data when we reached the universities for data collection, as we found by matching the study dates and timestamps of when these participants provided data. Thus, we imputed these 82 participants’ profession as “student.”

Although the participants were required to provide data at least once, we encouraged them to provide data as many times as possible. After excluding the 14 (0.47%) participants for whom our app could not collect any app usage events and the 2 (0.07%) participants who were not students, 2936 (99.46%) participants remained. Of them, 71 (2.42%) provided data at least twice. However, since there were few participants who provided data twice, in this study, we kept only the first time–provided data for the next steps of the analysis.

Since in this study, we will estimate the circadian rhythm, we followed previous studies to find out the minimum number of days required to estimate the circadian rhythm. Studies have suggested to have data of at least 1 day for estimating the circadian rhythm [81]. However, sensed data of 2 days can estimate the circadian rhythm sufficiently [82]. Researchers have also found that rhythmic features extracted based on the data of 2 days can predict more accurately physiological and mental changes [65]. Therefore, inspired by these previous studies, we excluded 34 (1.15%) participants who had app usage data of less than 2 days.

**Figure 2:**
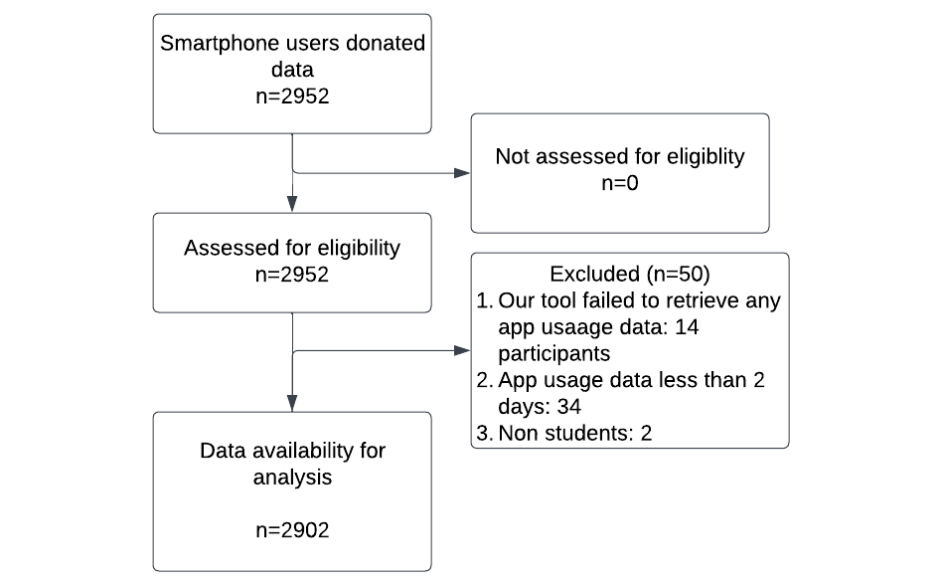
STROBE (Strengthening the Reporting of Observational studies in Epidemiology) flowchart.

**Figure 3:**
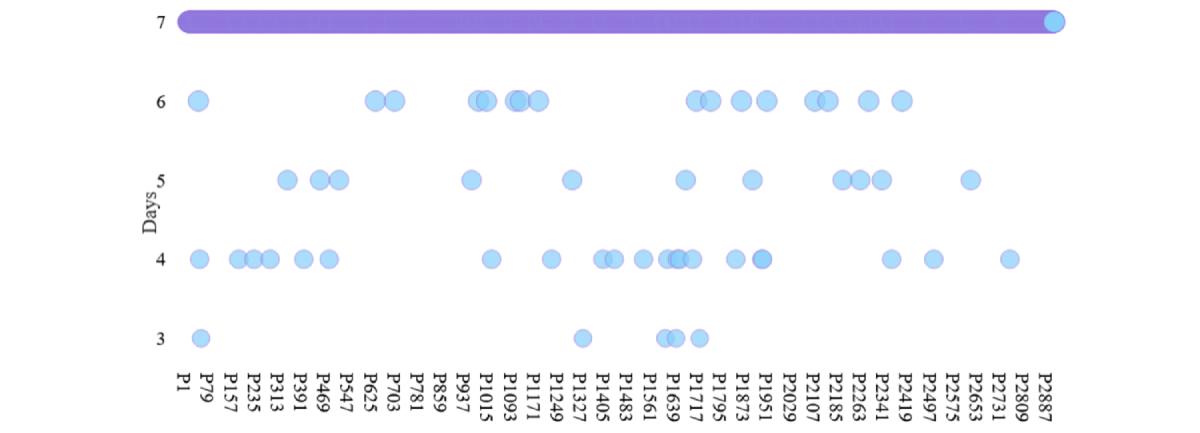
Number of days’ data that each participant has for the final analysis. P: participant.

Finally, after excluding 2 nonstudents, 14 students without any app usage data, and 34 students having app usage data of less than 2 days, we were left with 2902 (98.31%) students’ app usage data (Figure 2), which will be analyzed in the next steps. In total, there are 24.48 million foreground and background app usage events of 24.84 million minutes. Of the 2902 participants, 2849 (98.17%) have app usage data of 7 days (Figure 3).

### Ground Truth Data to Measure Depressive Symptoms

We used the PHQ-9 (Table 1) [15], which is one of the most commonly used scales to assess depression [21]. In our study, we used the PHQ-9 translated into Bengali [83], which has been validated and is largely used in Bangladesh (eg, [8,78]). The PHQ-9 score of 10 has a sensitivity and specificity of 88% in identifying MDD [15]. Based on this cutoff score, we will categorize the participants as depressed if the PHQ-9 score is at least 10.

In a previous study [67], a person was categorized as having a depressive symptom if that symptom appeared for several days or more. However, we will consider the threshold more than half of the days since the National Institute of Mental Health (NIMH) and the World Health Organization (WHO) define a person as depressed if symptoms appear for a specific time frame, such as most days for a minimum of 14 days [10,11]. In this study, if a participant reports being bothered by a depressive symptom (eg, hopelessness) in the PHQ-9 for more than half of the days or nearly every day of the past 14 days, we will categorize that participant as having that depressive symptom.

**Table 1:** Depressive symptom for each item of the PHQ-9^a^.

Symptom number	Symptom in the PHQ-9
1	Little interest or pleasure in doing things
2	Feeling down, depressed, or hopeless
3	Trouble falling or staying asleep or sleeping too much
4	Feeling tired or having little energy
5	Poor appetite or overeating
6	Feeling bad about yourself or that you are a failure or have let yourself or your family down
7	Trouble concentrating on things, such as reading the newspaper or watching television
8	Moving or speaking so slowly that other people could have noticed or the opposite—being so fidgety or restless that you have been moving around a lot more than usual
9	Thoughts that you would be better off dead or of hurting yourself in some way

^a^PHQ-9: 9-item Patient Health Questionnaire.

### Extraction of Behavioral Markers

As we will test the rhythmicity where the time series data are used [36] and explore the rhythmicity of the app usage behavioral markers instead of exploring raw foreground and background app usage events, we will set a time frame of 15 minutes based on which each app usage behavioral marker will be extracted. However, to check the robustness of the findings, we will follow the same process to explore rhythmicity using 2 other time frames as well: 10 minutes and 20 minutes.

In addition to calculating the usage duration, frequency of launching apps, entropy based on duration and entropy based on frequency of launching apps, and app usage sessions [33], we will calculate the following behavioral markers based on which we will extract parametric and nonparametric rhythmic features: relative importance of app categories, personalized top n apps, and cousage of apps.

#### Relative Importance of App Categories

In pervasive health research, aggregated app usage data (eg, [44-46]) are widely used, where the individual app categories remain unexplored. In our previous studies [33,74], we found that app category–based features are more important than aggregated smartphone usage regardless of category. However, in those studies, features of an app category were extracted independently regardless of the usage behavior of other categories. As a result, the individual category itself may not provide higher information for the app categories that are less used but contain distinguishable markers. For instance, if a participant launches social media apps 100 times and health and fitness apps only 10 times, data from the health and fitness category may get lower importance. However, data from the health and fitness category can be more important since depressed students use apps that contain features for smoking prevention and body weight reduction, which may not be used more frequently but may contain enough information to be significantly different from those used by nondepressed students [34]. Hence, we will calculate the term frequency–inverse document frequency (TF-IDF), which is a widely used technique in natural language processing [84] where less frequent terms across documents can get more importance. To adapt TF-IDF in the context of app usage, we will use data from all time frames over all days. In each time frame f, the app usage sessions s will work as the set of documents, and in each session (ie, a document) j, the list of categories of the used apps will act as the words.

Let the set of documents of participant i in f be {D_ij_, D_ij+1_, . . . D_is_}, where 
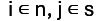
 , and n represents the number of participants. We will calculate TF-IDF_icj_ for each app category c based on the participant’s data of sessions s.

TF-IDF_icj_ = TF_icj_ × IDF_icj_,

where TF_icj_ = log[freq(c) + 1], IDF_icj_ = 
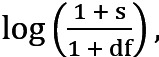
, freq(c) is the number of times app c was launched in session j, df is the number of sessions where c was used, and s represents the total number of sessions in f. After calculating TF-IDF_icj_ for each session, the mean TF-IDF value will be calculated for each category over the sessions of a time frame. Finally, using that mean value, we will extract the rhythmic features to understand how the relative importance of participant i in using a particular category c varied or remained constant over days and over the periods of a day and whether there was a rhythm in behavioral markers based on the TF-IDF.

To categorize the apps, we will follow relevant previous studies (eg, [30]) and the process in our previous studies [33-35,70,74], where we categorized the apps into more than 20 categories after exploring developers’ referred categories in Google Play Store and other app stores and discussing with graduate students of the computer science and engineering (CSE) department.

#### Personalized Top n Apps

We will calculate entropy based on the usage duration and the frequency of launching the top n apps, which can vary by student. To find the value of n, we will use the probability distribution and plot the probability of using apps on the y axis and the number of apps on the x axis. The point where the curve falls will be considered the threshold (ie, the value of n) to find the top n apps. To find the cutoff value, we will follow a previous study [46] that found cutoff values to exclude the participants having missing values.

#### Cousage of Apps

A significant portion of app usage consists of switching from one app to another [29]. One plausible reason for such behavior can be changing moods during app usage [85], and the users may do it to seek support, overcome negative emotions, etc. To quantify how participants switch from one app to another, we will calculate the cousage of apps, where the usage of 2 apps will be considered coapp usage if they are used in a single app usage session and also used consecutively. However, in a smartphone, there are many system apps that open automatically to support the function of another app. Users do not need to use those apps intentionally, and as a result, the inclusion of those apps may misrepresent behavior. In addition, to switch from one app to another, the user returns to the home screen of the smartphone, where the launcher app opens automatically. Considering the aforementioned issues, we will exclude these system apps and launcher apps before quantifying the coapp usage.

To find subtle differences in the variation in app usage patterns, we will build a network based on cousage, where each edge will present the cousage of 2 different apps. The weight of the edge will be calculated using point mutual information (PMI).



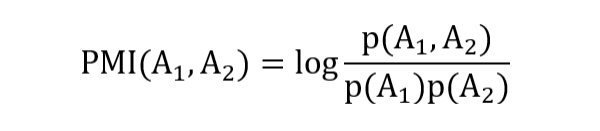



where p(A_1_, A_2_) represents the probability of A_1_ and A_2_ apps to consecutively appear in the same app usage session and p(A_1_) and p(A_2_) represent the probability of A_1_ and A_2_ apps, respectively, to appear in that session regardless of consecutiveness. Next, we will calculate the centrality and graph edit distance. The calculation of centrality will help us understand the most influential app in the network that is connected to the maximum number of nodes. The graph edit distance between 2 different sessions of the same time frame will inform whether the behavior differs. Finally, we will use the average data of each session of a time frame to explore the rhythmicity of the centrality and graph edit distance.

### Rhythm Analysis and Extraction of Rhythmic Features

The parametric cosinor method is one of the most widely used approaches to find out rhythmic parameters. However, cosinor analysis cannot find out the fragmentation in the rest-activity rhythm [86], which can be detected by extracting nonparametric rhythmic parameters, such as the IS. Therefore, to obtain a comprehensive picture of rhythms, we will conduct both parametric and nonparametric tests and extract the respective rhythmic features, as described later. In this study, to extract rhythms, instead of focusing only on all students’ data-based global models, we will also develop individual models and extract the rhythmic parameters for each participant. The main reason is that physiological data–based nonparametric rhythmic [87,88] and parametric rhythmic [89] parameters vary by the characteristics of people, and thus, there may also be a variation in parameters by the individual participant’s rhythm that is solely based on app usage data.

#### Dominant Period

In cosinor analysis, a cosine curve is fitted on the given period (eg, 24 hours) using the least squares method, where the model reduces the difference between observed and estimated values. However, cosinor analysis itself cannot estimate the best-fitting period [90]. Thus, we will empirically investigate through periodogram analysis and by setting the periods from 1 to 24. Later, the cosinor model will be developed for each given period (eg, 13 hours). The best-fitting period will be counted as the dominant period where the proportion of explained variance is the maximum.

#### Rhythm Detection

Cosinor analysis is a parametric method, and hence, we will first test normality [91]. Later, we will process the nonnormally distributed data through log transformation. Next, to find out whether a statistically significant rhythm of a participant exists, we will conduct a zero-amplitude test [36] by setting the significance level to .05. To find out whether an individual participant’s rhythm differs from the rhythm based on all participants, we will perform population mean cosinor analysis based on all the participants. In addition, we will develop a cosinor model for each participant and then calculate the average of the cosinor rhythmic parameters [90].

#### MESOR, Amplitude, and Acrophase

After developing the cosinor model, we will extract the following parametric rhythmic parameters for each participant: midline estimating statistic of rhythm (MESOR), amplitude, and acrophase. MESOR is the rhythm-adjusted mean [36], the amplitude presents the difference between the equilibrium position and the peak point of the rhythm oscillation [92], and the acrophase presents the timing of the high values recurring in each cycle of the rhythm [36]. Since the amplitude can present the strength of the rhythm [93], while comparing the diurnal (12-hour period) and circadian (24-hour period) rhythms to determine which rhythm has more strength, we will compare the amplitude. Moreover, we will compare the coefficient of determination as it presents how well a model fits in a given period.

#### Interdaily Stability and Intradaily Variability

A person’s mental state has a relation with the IS and the IV. For example, patients with bipolar disorder have less IS and higher IV [94]. Similar patterns may be found in the IS and the IV based on app usage data, and we will calculate the IS and the IV, based on a previous study [86] on actimetry.



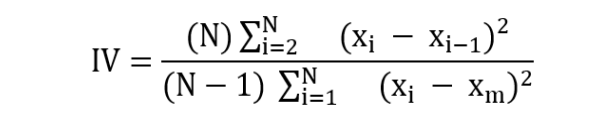



Here, x_i_ is the behavioral marker’s value at a specific time frame, x_m_ is the mean value of the same behavioral marker in all time frames, and N is the number of time frames.



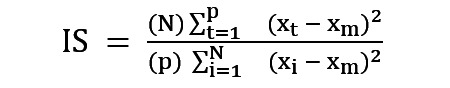



Here, x_i_ is the behavioral marker’s average value in that time frame over days and p is the number of time frames per day. The range of the IV value is 0-2 and that of the IS value is 0-1 [95]. The higher the IS, the greater the stability, as the name implies. However, the higher the IV, the higher the fragmentation in the rhythm. If a difference remains, for example, if someone sleeps during the daytime and keeps waking up at nighttime, their IV will be higher [86].

#### M10, L5, and Relative Amplitude

M10 presents the mean value of the most active consecutive 10 hours and provides information about the diurnal activity. Active persons have a higher M10 [86], and a lower M10 can be associated with exercise reduction [86] and also with a negative mental state [86,96]. L5 presents the mean value of the least active 5 consecutive hours of the day. It is a measure of nocturnal activity, and the higher L5 may represent activity during the rest cycle [86]. After calculating M10 and L5, we will calculate the RA = (M10 – L5)/(M10 + L5). As can be understood from the formula of RA, the RA is actually the normalized difference between M10 and L5. The larger the difference between L5 and M10, the larger the RA. People with psychological problems can have a lower RA [65,86].

### Statistical Analysis

To explore whether there is any relation between depressive symptoms and app usage rhythmic features, we will perform binomial logistic regression. As having a variance inflation factor (VIF) of more than 5 can create a biased regression model [97], we will eliminate the variable if the VIF of any variable goes beyond 5.

### ML Model Development

#### Participant Similarity and Development of Personalized Models

Most of the existing models (eg, [33,45,46,74]) to predict depression use training data to predict the outcome regardless of the characteristics of the participant whose class will be predicted. These models may have issues regarding generalizability since all individuals have unique characteristics that may not be captured in one-size-fits-all models (ie, 1 set of training data to predict the depression of all test participants). Indeed, through empirical investigation, it has been found that the personalized model performs better than the one-size-fits-all model [98,99]. The one-size-fits-all or global model is likely to capture the general or the “average” characteristics of the participants for the prediction due to which the model may perform well only on the “average” participants and may not work in the case of the participants whose behavior deviates from the “average” participants [100]. However, the personalized model is trained dynamically for each participant, which can facilitate finding the most relevant set of features to predict the outcome of the test participant [98]. Therefore, a personalized model may perform better in predicting depressive symptoms since each of the depressed students has a unique app usage signature [34] and depressed students have statistically significantly different smartphone usage behaviors than nondepressed students [35], as we found in our previous studies [34,35].

In our app to predict depressive symptoms, we will use each participant i’s rhythmic parameters based on each of the app usage behavioral markers of m_i_ to calculate the cosine similarity with all other participants n – 1.



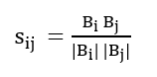



Here, B_i_ and B_j_ represent the vectors 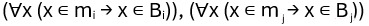 based on the rhythmic parameter sets m_i_ and m_j_ of participants i and j, respectively. The cosine similarity s_ij_ will be close to 1 when there is a higher similarity between participants i and j. After finding the similarities between participant i and all other participants, we will use the most similar N participants to train the MTL framework for participant i. The value of N will be decided empirically through a search on the values 200, 250, 300, and 350.

#### MTL Framework Development and Validation

Although some similar tasks remain, the development of the model through the MTL framework can facilitate improving the performance of the models through information sharing. In addition, if different models are developed for different tasks, then the system can be resource inefficient. In a previous study [65], researchers used MTL to develop systems for predicting the symptoms of schizophrenia. However, in that system, all symptom prediction tasks were considered the same. In reality, all the tasks included in a model do not help one another improve performance [68]. Therefore, we will find the similarities among the symptoms and use similar symptom prediction tasks in a model, while other similar symptom prediction tasks will be in another model. To group the tasks, we will calculate the correlation coefficients among the symptoms. Symptoms that are highly (coefficients>0.7), moderately (0.4≤coefficients≤0.7) [101], and less than moderately correlated or not correlated will be kept in 3 different groups.

While developing the MTL framework, we will use the hard parameter-sharing technique since in this approach, the model can find a common representation to capture all the tasks that can reduce the potential risk of overfitting [102]. Combining multiple loss functions leads to promising performances [103]. In our study, we will use a weighted loss of the hinge and cross-entropy. However, we will not use a fixed weight. Instead, we will tune the weight using the Bayesian search optimization technique, which selects the next parameter based on the performance of the previously selected one.

To validate the framework, we will use the nested cross-validation (CV) method since this approach is found to have a generalizable performance compared to K-fold CV [104]. In the outer loop, we will use the leave-one-out cross-validation (LOOCV) method, and in the inner loop, we will use a 10-fold CV, where in each interaction, 9 folds will be used for tuning the hyperparameters and the remaining 1 fold will be used for validation. We are aware of the fact using the LOOCV will increase the time complexity since there are 2902 participants and we will be developing a personalized model for each of them. However, we chose to use the LOOCV because it will work like the real-world scenario, where the model will predict a single participant’s depressive symptoms at a time. In addition, this process will help in personalizing the model, where we will use only those participants in model training who are similar (in terms of app usage behavior without using any information about depressive symptoms) to the student for whom the model will predict the symptoms, as discussed in detail in the *Participant Similarity and Development of Personalized Models* section. During model development, we will maximize the balanced accuracy as it is based on sensitivity and specificity, and having a higher balanced accuracy can lead to higher precision and F_1_-score values.

To understand the robustness of the proposed MTL framework, we will compare it with the performance in the following approaches:

- Comparison with STL-based models: We will compare the performance of the proposed personalized MTL framework with that of STL models. This will resemble the approach presented in a previous study [67], where each symptom was considered a single task. To develop the STL model, we will use ML algorithms, such as the random forest (RF), support vector machine (SVM), decision tree (DT) [105], and logistic regression [106], which are widely used in medical informatics, as shown in systematic reviews [105,106].
- Comparison with the nonpersonalized MTL framework: Since we expect that personalization may provide better performance, as discussed in the *MTL Framework Development and Validation* section, we will compare the performance of the personalized MTL framework with that of a nonpersonalized MTL framework, where we will use n – 1 participants’ data for training instead of using a personalized subset of data.
- Comparison with the MTL framework without grouping tasks: To understand how grouping tasks based on similarity impact performance, we will compare the performance of the MTL framework with and without grouping tasks.

## Results

As mentioned earlier, after applying inclusion criteria, we kept the data of 2902 (98.31%) of 2952 students for analysis, with the data of 24.48 million app usage events, and 7 days’ app usage of 2849 (98.17%) students.

**Figure 4:**
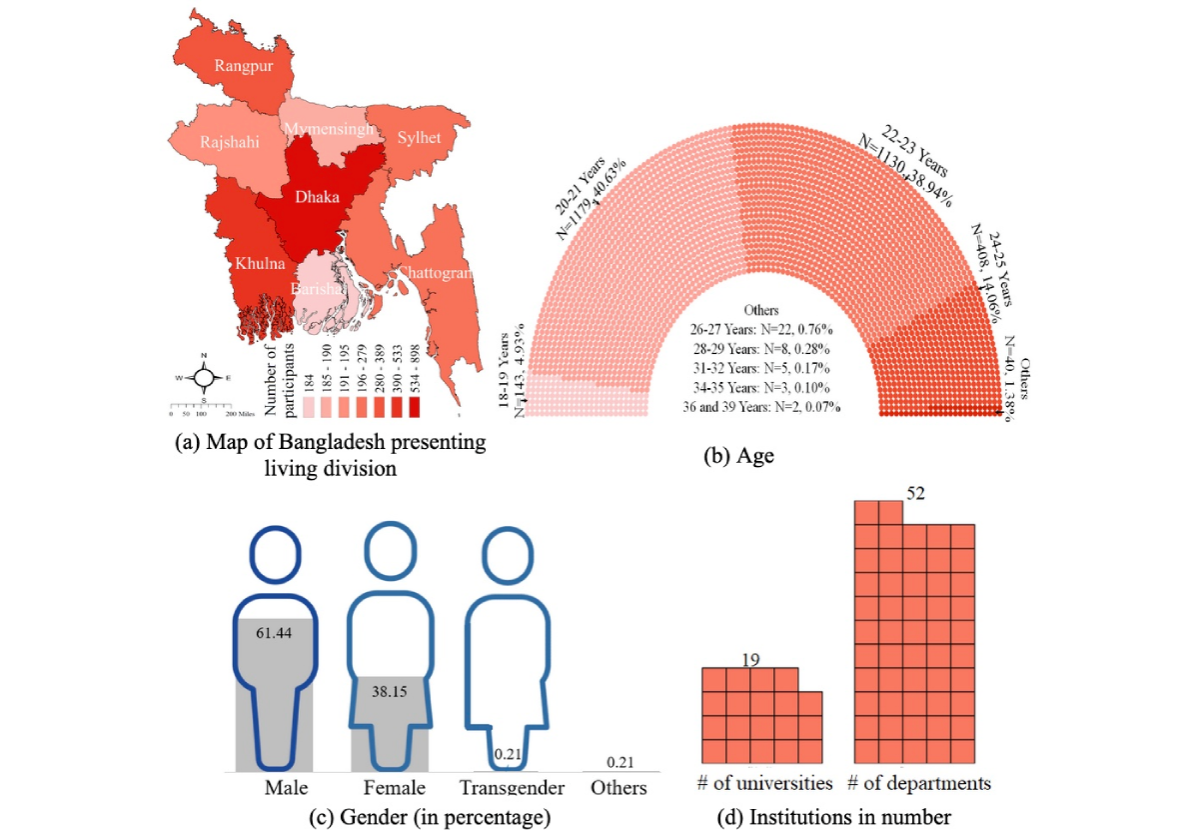
Participants’ demographic characteristics: (a) map of Bangladesh presenting living divisions, (b) age, (c) gender, and (d) number of different institutions.

Figure 4 shows the findings regarding participants’ demographic characteristics. The participants were from the 8 divisions of Bangladesh (Figure 4a). Most participants (n=887, 30.56%) were from the Dhaka division, which also reflects the fact that the majority of university students of Bangladesh reside in this division. The participants’ age varied from 18 to 39 years, and 2309 (79.57%) participants were aged 20-23 years (Figure 4b). Of the 2902 participants, 1107 (38.15%) and 1783 (61.44%) were female and male participants, respectively (Figure 4c). There were 2430 (83.74%) and 472 (16.26%) students from public and private universities, respectively. The participants belonged to 19 universities (Figure 4d), including specialized universities, studying the following subjects: agriculture, engineering, and textiles. They also belonged to 52 different departments, including arts (eg, Department of Sculpture), business (eg, Department of Management Studies), engineering (eg, Department of Petroleum & Mining Engineering), science (eg, Department of Botany), textiles (eg, Department of Apparel Engineering), public health, and law faculties.

In the remaining part of the study, we will work on rhythm detection, rhythmic feature extraction, and MTL framework development. We expect to publish our findings by June 2024.

## Discussion

### Significance

By using the data set constructed through a countrywide study on 2902 students having over 24 million app usage events, we will explore whether there is a statistically significant rhythm based on the different app usage behavioral markers. We hypothesize that app usage behavioral markers, such as the relative importance of an app category, have rhythmic patterns with reproducible waveforms because, like physiological data, the markers vary depending on factors such as the time of day [29-31]. In addition, since rhythmic features based on physiological [39,107] and activity [108] data have potential applications in problems such as determining which participants have a higher risk of disease [107], determining sedentary behavior [39], and finding subtle changes in detecting COVID-19 [107], an in-depth exploration of app usage marker–based rhythms may show an alternative source of data to understand the rhythms in human life. App usage marker–based rhythms have the possibility to be used for different purposes. For example, a statistically significant relation between the rhythmicity of app usage and depressive symptoms can create the possibility of using these rhythmic features for an intervention to mitigate depression.

In addition, by predicting depressive symptoms, our study will extend the findings of previous studies since most studies (details in a recent systematic review [43]) in the pervasive health area have developed classification models (eg, to classify depressed and nondepressed individuals) where the complexity of the psychological problem of depression may be lost. For instance, a participant with a PHQ-9 score of 10 has moderate depression [15], and a score of 10 can result from different combinations of the subscores of the 9 symptoms in the PHQ-9. As a result, by classifying participants into a few groups based on the overall score on a scale, it is not possible to precisely determine the depressive symptoms that bother a student. However, it is important to know since each depressive symptom (eg, symptoms in the PHQ-9 [15]) presents a unique phenomenon (eg, anhedonia, sleep disturbance, suicidal ideation) [109]. Therefore, depending on our proposed personalized MTL framework’s performance based on real-time data, the proposed app can contribute to early diagnosis of depressive symptoms and precise understanding of a depressed student, which, in turn, may contribute to mitigating depression prevalence.

Our previous pilot studies in Bangladesh on the relation of app usage with depression [35,73] and loneliness [75], classifying depressed and nondepressed students [33] and with and without loneliness [74], showed promising models solely based on resource-insensitive [33] app usage behavioral markers. Incorporating app usage rhythmic features and also the MTL framework by leveraging the similarities among the symptoms’ prediction tasks so that tasks do not hurt one another’s performance may help researchers and developers in developing more robust models to predict the symptoms of psychological problems solely through app usage data. In addition, our app’s reliance on data retrieved from a smartphone within 1 second [33] may also make it feasible in LMICs since smartphones are more affordable [42] compared to wearables [41] that are usually used to obtain physiological data and extract rhythmic features.

### Strengths

The median sample size of previous studies that classified or predicted depression was 58, and none of the studies that developed computational models for prediction tasks had a sample size of over 500, as shown in a recent systematic review [21]. However, we constructed a large data set comprising 2902 students. In addition, the participants of our study are from all 8 divisions of Bangladesh, from both public and private universities and 52 different departments. To the best of our knowledge, this is the largest data set containing data on both app usage and depressive symptoms. Considering these facts, our findings based on the proposed methods may be generalizable, may be robust enough to be impactful in the real world, and may contribute significantly to advancing the knowledge in mobile and pervasive health research areas.

To the best of our knowledge, this will be the first study to explore in depth rhythms based on different app usage behavioral markers, which can create an opportunity to find an alternative source of data to understand the rhythms of daily life without depending on physiological data–based rhythms, which are usually retrieved by costly wearables.

In our recent work based on app usage [33], our developed app had higher performance in predicting depression than the existing systems based on app usage as well. Through feature analysis (for details, please see Ref. [33]), we found that our newly explored behavioral markers (eg, ratio of the hamming distance [33]) were more important than the features used in previous studies. That being said, performance varies depending on the behavioral markers used in a model. Hence, the novel behavioral markers (eg, relative importance of app categories, cousage of apps) we presented in this protocol that were not explored in previous pervasive health research have significance. In addition, to predict depressive symptoms, we will develop a personalized MTL framework. Although an MTL framework has been developed in some previous studies (eg, [65]) to predict a person’s mental state, our study will add to this knowledge by showing the performance of a personalized MTL framework.

### Limitations

Following previous studies [65,81,82], to analyze rhythms, we have included 2902 participants in this study who had app usage data of at least 2 days and most of whom (n=2849, 98.17%) had app usage data of 7 days. Data of more than 7 days will help us better understand the stability of the rhythms, the rhythm disruption over weeks, and its potential effect on depressive symptoms’ appearance. However, we believe our study can work as a primary cursor for future studies to further explore app usage rhythms.

Although our study includes over 2900 students from different divisions of Bangladesh, our proposed app may not be generalizable to every student since behavior varies depending on many factors, such as season, region, [22], and socioeconomic status [23]. We recommend future studies to include participants based on more factors that can impact behavior. Moreover, in our study, although we have included participants from all 8 divisions of Bangladesh, we could not include participants from all 64 districts of the 8 divisions. In addition, including more participants from rural areas could have potential to obtain a more reliable picture of the students’ behavior, which, in turn, can be useful to develop a better app.

### Conclusion

Predicting depressive symptoms accurately could help in better diagnosis of depression and in taking appropriate steps accordingly. However, existing models regarding symptom prediction are limited by various issues, including low performance (eg, specificity is around or below 60% for most symptoms). Our proposed approach to explore rhythmic features from app usage behavioral markers and the development and validation of the MTL framework through our constructed large-scale data set may provide new insights into rhythms and higher performance in predicting depressive symptoms.

## Abbreviations

AI: artificial intelligence
CV: cross-validation
IS: interdaily stability
IV: intradaily variability
LMIC: low- and middle-income country
LOOCV: leave-one-out cross-validation
MDD: major depressive disorder
MESOR: midline estimating statistic of rhythm
ML: machine learning
MTL: multitask learning
PHQ-9: 9-item Patient Health Questionnaire
PMI: point mutual information
RA: relative amplitude
STL: single-task learning
TF-IDF: term frequency–inverse document frequency
VIF: variance inflation factor

## Data Availability

Since app usage data are sensitive, making the data publicly available can raise different data privacy and safety concerns (eg, reidentification of the participants [34]). Thus, we do not plan to upload the data to any public data repositories. However, the data can be accessed by sending a reasonable request to the corresponding author.
